# Tissue oxygenation as a target for goal-directed therapy in high-risk surgery

**DOI:** 10.1186/cc13338

**Published:** 2014-03-17

**Authors:** PA Van Beest, JJ Vos, M Poterman, AF Kalmar, TW Scheeren

**Affiliations:** 1 University Medical Center Groningen, the Netherlands

## Introduction

Tissue hypoxia occurs frequently during surgery and may contribute to postoperative organ dysfunction [1]. We hypothesised that intraoperative optimisation of tissue oxygenation reduces postoperative complications and evaluated the feasibility of the optimisation protocol used.

## Methods

We randomised 50 high-risk patients who underwent major abdominal surgery. Tissue oxygenation was monitored at the thenar eminence using near-infrared spectroscopy. All patients were treated according to a standard care algorithm. In addition, patients in the intervention group received dobutamine if necessary to keep tissue oxygenation ≥80%. Data were recorded continuously and complications were recorded during the hospital stay with a maximum of 28 days.

## Results

**Figure 1:**
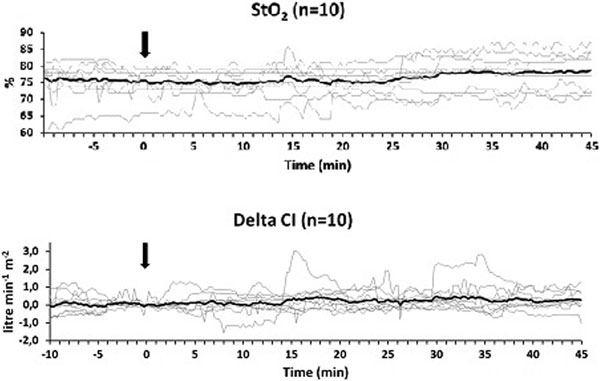
**Evolution of individual patient values (thin lines) and the average values (thick lines) of the tissue oxygenation (StO_2_) and the relative change of cardiac index (CI), delta CI. All graphs are synchronised at the moment of the first dobutamine administration (time = 0; arrow)**. Values are shown from 10 minutes before dobutamine administration until 45 minutes after.

The number of complications tended to be lower in the intervention group (11 vs. 20). Eleven patients in the intervention group had no complication, versus seven in the control group. There was no significant difference between groups in length of stay in ICU or in hospital. Administration resulted in a 5% increase of tissue oxygenation. The cardiac index increased 0.3 (0.0 to 0.6) l/minute/m^2 ^(Figure 1). The overall protocol adherence was 94%.

## Conclusion

Intraoperative optimisation of tissue oxygenation will potentially result in better outcome after high-risk abdominal surgery. The protocol used may be considered feasible for clinical practice.
